# Site Specific Effect of Tobacco Addiction in Upper Aerodigestive Tract Tumors: A Retrospective Clinicopathological Study

**DOI:** 10.1155/2014/460194

**Published:** 2014-11-06

**Authors:** Ashok Kumar, Amita Sharma, Babita Ahlawat, Sonam Sharma

**Affiliations:** ^1^Department of ENT, SHKM Govt Medical College, Nalhar, Mewat, Haryana, India; ^2^Department of Dentistry, SHKM Govt Medical College, Nalhar, Mewat, Haryana, India; ^3^Department of Pathology, Vardhman Mahavir Medical College & Safdarjung Hospital, New Delhi, India

## Abstract

An institutional study was carried out in 102 patients to investigate the site specific effect of addictions, that is, tobacco smoking and tobacco chewing (smokeless), both independently and synergistically in development of malignancies in upper aerodigestive tract through retrograde questionnaire. The histopathologically proven cases were interviewed regarding different forms of addictions followed by clinical examination and investigations for grading (according to Modified Broadmann's method) and TNM staging (according to UICC) according to the tumor site. Statistical analysis was done by Pearson test. Out of all proven cases of cancers, 29.4% were only tobacco chewers (smokeless), 25.5% were only smokers, 42.2% were having both types of tobacco addictions (smoke and smokeless), and only 2.9% were having no addiction. Out of only tobacco chewers (smokeless), 83.3% were of oral cavity cancers, 6.7% were of oro- and hypopharynx and the rest were of others. Among only smokers, 69.2% cases were of laryngeal and oro- and hypopharynx as compared to 11.5% of oral cavity cancers (nearly 6 times). Tobacco (smokeless) chewing is associated with oral cancers whereas tobacco smoking is associated with laryngeal and hypopharyngeal carcinoma. Both smoking and smokeless tobacco act in synergy with each other.

## 1. Introduction

Head and neck squamous cell carcinoma (HNSCC) is the sixth most common cancer with annual incidence of 500,000 cases worldwide. HNSCC is a heterogeneous group of cancers, with usually a poor prognosis in patients [1]. While head and neck cancer is relatively rare in the United States, it is a more significant entity in some parts of the developing world. The vast majority of head and neck malignancies are squamous cell carcinomas of the upper aerodigestive tract (UADT), that is, the oral cavity, the oro- and hypopharynx, and the larynx. In the United States alone, approximately 28,000 men and 12,000 women are diagnosed with HNSCC each year, 3.2% of all newly diagnosed cancers; the disease also accounts for 2.1% of cancer-related deaths [2]. Overall 57.5% of global head and neck cancers occur in Asia, especially in India accounting for 30% of all cancers [3, 4]. HNSCC is associated with severe disease and treatment related morbidity and has a 5-year survival rate of approximately 50%; this rate has not improved in more than 2 decades [5]. Approximately 90% of HNSCCs occur after exposure to tobacco and/or alcohol. Emerging information suggests that one's genetic background and exposures apart from tobacco and alcohol contribute to the risk of HNSCC as well. Tobacco use and alcohol intake are the major risk factors for development of HNSCC [6]. Therefore, this study was aimed at investigating the site specific effect of tobacco addiction, that is, tobacco chewing (smokeless) and smoking tobacco, both independently and synergistically in development of malignancies in upper aerodigestive tract.

## 2. Material and Methods

The study was carried out on 102 patients in the Department of Otorhinolaryngology and Head and Neck Surgery of a Medical College and Hospital in Madhya Pradesh, India, over a period of one year. Histopathologically proven cases of malignancy in upper aerodigestive tract of all age groups constituted the material for this study. The patient was made comfortable and a detailed clinical history in each case with special reference to age of onset, duration of illness, symptoms, occupation, and personal history, past history of syphilis, tuberculosis, and septic foci, was taken. The history of various addictions was elicited in details regarding type, dose, duration, and mode of intake. All the patients were subjected to physical examination both general and local. Examination of ear, nose, and throat region was done which consisted of inspection and palpation of oral cavity and oropharynx; examination of postnasal region with posterior rhinoscopy mirror; indirect laryngoscopy; direct laryngoscopy, nasopharyngoscopy, and oesophagoscopy under local or general anesthesia were done to localise the site of the tumour. Routine laboratory investigations along with radiological examination of chest; systemic examination including cardiovascular, central nervous, respiratory systems, and gastrointestinal tract was done. Fine needle biopsy (FNAB) from lymph nodes and other suspicious swellings of the region, if involved, was done. Histological examination of biopsy taken from other suspicious sites and secondaries in lymph nodes was done. The histopathological grading of the tumor was done according to Modified Broadmann's method as Grade I: well differentiated carcinoma, Grade II: moderately differentiated carcinoma, Grade III: poorly differentiated carcinoma, and Grade IV: undifferentiated carcinoma. TNM staging was done according to UICC classification (International Union against Cancer, 1997) [7]. The classified diagnosis was made according to the ICD-9 coding [8]. Statistical analysis was done by Pearson test.

## 3. Results

Out of total 102 cases of upper aerodigestive tract tumours, proven by histopathological reports, 85 (83.4%) were males and 17 (16.6%) were females; M : F ratio was sent to be 5 : 1. The mean age of presentation was 51.13 (±13.94) years for males and 60.71 (±12.02) years for females.

Table 1 shows correlation between use of smokeless tobacco chewing and smoking as independent and synergistic factors in relation to classified diagnosis. The present study shows only 3 cases out of 102 did not use tobacco in any form, that is, 2 cases of Ca buccal mucosa and 1 case of Ca lip. The remaining 99 cases, that is, 97% of cases with UADT tumours, have positive history of tobacco addiction in one form or the other and hence have a statistical significant etiological role. Among 99 cases 30 (29.4%) used to chew tobacco (smokeless), 26 (25.5%) used to smoke tobacco, and 43 (42.2%) used tobacco in both forms.

As an independent factor tobacco chewing (smokeless) is found in 12 (40%) cases of Ca buccal mucosa, 9 (30%) cases of Ca tongue, and 3 (10%) cases of Ca lower alveolus and 1 each in Ca oropharynx Ca oesophagus, Ca lip, Ca larynx, Ca hypopharynx, and Ca ethmoidal sinus.

As an independent factor tobacco smoking is found in 13 (50%) cases of Ca hypopharynx, 4 (15.5%) cases of occult primary and Ca oropharynx, 2 (7.7%) cases of Ca floor of mouth, and 1 (3.8%) case each of Ca larynx, Ca tongue, and Ca oesophagus, but no case of Ca buccal mucosa, Ca lower alveolus, and Ca lip has history of only tobacco smoking.

The synergistic effect of both modes (tobacco chewing and smoking) is found in 11 (25.6%) cases of Ca buccal mucosa, 9 (20.9%) cases of Ca tongue and 8 (18.6%) cases of Ca oropharynx, 5 (11.6%) cases of Ca hypopharynx, 3 (7%) cases of Ca lower alveolus and Ca larynx each, and 1 (2.3%) case each of Ca floor of mouth, Ca oesophagus, Ca lip, and occult primary. No case of Ca ethmoidal sinus was found to have history of tobacco addiction by both modes.

## 4. Discussion

In the present study, UADT tumours were observed to be more common in males with a M : F ratio as 5 : 1. According to age group head and neck cancer were more common in 4th to 6th decade. Thus it is observed that males were presenting a decade earlier than females. Teshima et al. [9], Hiranandani [10], Issing et al. [11], Kim et al. [12], and Ologe et al. [13] found similar higher incidence in age group of 5th and 6th decade and male : female ratio around 4 : 1. Fried et al. [14] found higher incidence in age group of 6th and 7th decade, M : F as 2 : 1. Thus it can be concluded from the present study that male preponderance, mean age of presentation was correlating with other studies. It was also seen during comparison that in the course of time the mean age of presentation has decreased in comparison to older studies; this shows the changing pattern of head and neck cancers. The cause can be explained by the fact that awareness in population and availability of medical facility could be the reasons for early presentation at hospital. The environmental factors and increased addiction habits were also contributing factors.

In our study, majority of cases were addicted to tobacco 97% whereas 8% had alcohol as additional addiction and only 3% cases were totally not addicted to any addiction. This describes tobacco addiction as a causal factor in carcinogenesis of UADT tumour. This is comparable with studies by Rothman [15].

In our series the cases with tobacco chewing were observed at higher risk for carcinoma in oral cavity than other sites, whereas tobacco smoking has higher risk for carcinoma hypo- & oropharynx and carcinoma larynx as compared to other sites. This was in accordance with observations of Williams and Horm [16], Jayant et al. [17], Nandakumar et al. [18], and Sankaranarayanan et al. [19].

It was indirectly proved that chewing tobacco has strong correlation with carcinoma oral cavity as compared with tobacco smoking. The present study is in accordance with study by Znaor et al. [20], which was conducted in Chennai and Trivandrum, South India. Tobacco chewing emerged as the strongest risk factor for oral cancer while strongest risk factor for pharyngeal and laryngeal cancers was tobacco smoking, Sankaranarayanan et al. [19], using average attributable risks weighted by numbers of cases reported that 19% of cases could be prevented by eliminating smoking alone, 73% could be prevented by elimination of tobacco chewing alone, and 85°% could be prevented by elimination of both habits. Nandakumar et al. [18] also states that a distinction of anatomic subsites in relating risk factors is important. Due to embryologic and anatomic development and also because in tobacco chewing the areas of oral cavity are exposed to a greater degree than the oro- & hypo-pharynx. Jayant et al. [17] also concluded that the aetiologic fraction due to tobacco chewing is high for cancers of the oral cavity and hypopharynx and etiologic fraction due to smoking is high for cancers of the oropharynx and larynx but lower for cancers of other sites. The values of the indices of synergy studied between tobacco chewing and smoking for cancers of the oral cavity, oropharynx, hypopharynx, larynx, and oesophagus have shown that, at each of the above sites, tobacco smoking and chewing act synergistically, not independently.

Thus it is evident from the present study that tobacco chewing (smokeless) has etiological correlation with carcinoma of oral cavity (i.e., Ca buccal mucosa > Ca tongue > Ca lower alveolus), whereas tobacco smoking is etiologically related to Carcinoma pharyngeal and laryngeal region (i.e., hypopharynx > oropharynx > larynx). As a combined effect of both modes chewing and smoking the synergistic role presents on cases of Ca buccal mucosa > Ca tongue > Ca oropharynx > Ca hypopharynx, followed by cases of Ca lower alveolus and Ca larynx.

## 5. Conclusion

In an attempt to evaluate the role of tobacco addiction in development of tumour in different sites of upper aerodigestive tract, we conclude that tobacco is the most prevalent addiction and has an etiological role as a risk factor in cases of upper aerodigestive tract tumours that can be used for both chewing and smoking. Tobacco chewing (smokeless) is associated with oral cavity tumours whereas tobacco smoking is associated with hypo- & oropharyngeal and laryngeal carcinomas. Both tobacco chewing and smoking act in synergy with each other at sites of direct exposure, that is, oral cavity, oro- and hypopharynx. Therefore, a drive should be generated to create awareness against tobacco addiction so as to make our society cancer-free.

## Figures and Tables

**Table 1:** Correlation between use of smokeless tobacco and tobacco smoking with classified diagnosis.

Classified diagnosis	ICD code	No tobacco (%)	Only smokes (%)	Only tobacco chewers (%)	Both (%)	Total (%)
Ca. buccal mucosa	ICD-145	2 (8.0) (66.7)	0	12 (48.0) (40.0)	11 (44.0) (25.6)	25 (100.0) (24.5)

Ca. floor of mouth	ICD-144	0	2 (66.7) (7.7)	0	1 (33.3) (2.3)	3 (100.0) (2.9)

Ca. hypopharynx	ICD-148	0	13 (68.4) (50.0)	1 (5.3) (3.3)	5 (26.3) (11.6)	19 (100.0) (18.6)

Ca. larynx	ICD-161	0	1 (20.0) (3.8)	1 (20.0) (3.3)	3 (60.0) (7.0)	5 (100.0) (4.9)

Ca. lip	ICD-140	1 (33.3) (33.3)	0	1 (33.3) (3.3)	1 (33.3) (2.3)	3 (100.0) (2.9)

Ca. lower alveolus	ICD-143	0	0	3 (50.0) (10.0)	3 (50.0) (7.0)	6 (100.0) (5.9)

Ca. oesophagus	ICD-150	0	1 (33.3) (3.8)	1 (33.3) (3.3)	1 (33.3) (2.3)	3 (100.0) (2.9)

Ca. oropharynx	ICD-146	0	4 (25.0) (15.4)	1 (8.3) (3.3)	8 (66.7) (18.6)	13 (100.0) (12.8)

Ca. tongue	ICD-141	0	1 (5.3) (3.8)	9 (47.4) (30.0)	9 (47.4) (20.9)	19 (100.0) (18.6)

Ca. ethmoidal sinus	ICD-160	0	0	1 (100.0) (3.3)	0	1 (100.0) (1.0)

Occult primary	ICD-196	0	4 (80.0) (15.4)	0	1 (20.0) (2.3)	5 (100.0) (4.9)

Total		**3** (**2.9**) (**100.0**)	**26** (**25.5**) (**100.0**)	**30** (**29.4**) (**100.0**)	**43** (**42.2**) (**100.0**)	**102** (**100.0**) (**100.0**)

Mean ± SD, Pearson correlation coeff.		3.90 ± 3.8, 1.00^*^	2.36 ± 3.82, 1	2.72 ± 3.97, −.282	3 ± 3.80, 1	—

^*^Correlation is significant at .01 level of significance.
